# The deubiquitylase USP2 maintains ErbB2 abundance via counteracting endocytic degradation and represents a therapeutic target in ErbB2-positive breast cancer

**DOI:** 10.1038/s41418-020-0538-8

**Published:** 2020-04-23

**Authors:** Jinrui Zhang, Shuyan Liu, Qiong Li, Yulin Shi, Yueguang Wu, Fang Liu, Shanshan Wang, Mohamed Y. Zaky, Waleed Yousuf, Qianhui Sun, Dong Guo, Taishu Wang, Yingqiu Zhang, Yang Wang, Man Li, Han Liu

**Affiliations:** 1 Second Affiliated Hospital, Institute of Cancer Stem Cell, Dalian Medical University, Dalian, China; 2 Molecular Physiology Division, Department of Zoology, Faculty of Science, Beni-Suef University, Beni-Suef, Egypt; 3 Department of Oncology, Second Affiliated Hospital, Dalian Medical University, Dalian, China

**Keywords:** Deubiquitylating enzymes, Ubiquitylation, Cancer

## Abstract

ErbB2 overexpression identifies a subclass of breast cancer as ErbB2-positive that is frequently associated with poor prognosis. Current ErbB2-targeted therapies have profoundly improved patient outcomes, but mutations occurring in *ErbB2* have been shown to confer drug resistance. Induction of ErbB2 degradation was proposed as an intriguing strategy to battle with ErbB2-positive breast cancer and reduced mutation-incurred drug resistance. Although multiple HSP90 inhibitors have been demonstrated to effectively trigger ErbB2 degradation, none succeeded in the clinical evaluations. To develop novel ErbB2-targeting strategies, we investigated the endocytic degradation and reversible ubiquitylation of ErbB2 in breast cancer. In this study, we reveal that HSP90 inhibition leads to efficient ubiquitylation and endocytic degradation of ErbB2 through the canonical endo-lysosomal route. USP2 associates with internalized ErbB2 and prevents its lysosomal sorting and degradation via exerting deubiquitylase activity. Accordingly, the USP2 inhibitor ML364 is capable of inducing ErbB2 ubiquitylation and accelerating its turnover. ML364 potentiates the pro-degradation effects of HSP90 inhibitors on ErbB2 and hence sensitizes ErbB2-positive breast cancer cells to HSP90 inhibition. The combination of USP2 and HSP90 inhibitors effectively restrains ErbB2-positive breast cancer xenograft growth in vivo. Based on these observations, we conclude that USP2 safeguards ErbB2 surface levels by antagonizing its ubiquitylation-mediated endocytic degradation, which can be exploited to design novel therapeutic strategies against ErbB2-driven malignancies as combinatorial treatment with HSP90 inhibitors.

## Introduction

Receptor tyrosine kinases (RTK) comprise a group of transmembrane proteins that transmit extracellular signals to regulate a diverse array of intracellular signaling circuitries, which have been associated with essential events during development, adult homeostasis, and human diseases [1]. The ErbB family of RTK has been closely associated with the development and progression of a number of malignancies, with two members EGFR and ErbB2 already proved as efficient therapeutic targets that received FDA approvals [2–4]. In particular, ErbB2 overexpression categorizes a subclass of breast cancer called ErbB2/Her2-positive, which represents approximately 20–30% among all type breast malignancies and is generally associated with poor prognosis [5]. Correspondingly, ErbB2 targeting has proved an effective approach in the battle against ErbB2-positive breast cancers, and current therapies approved by the FDA include ErbB2-directed antibodies and small molecule tyrosine kinase inhibitors [3]. Nevertheless, the seemingly unavoidable resistance to the therapeutic agents against ErbB2 was acquired by cancer cells in most cases through diverse mechanisms, which include mutations occurring in *ErbB2* that abrogate antibody or inhibitor binding [6–8].

In the endeavor to restrain development of drug resistance and further improve patient outcomes, alternative ErbB2-targeting strategies have been proposed [9]. One compelling approach is suggested to suppress ErbB2-positive cancer growth via promoting ErbB2 degradation. It is conceivable that this strategy will likely eliminate resistance incurred by ErbB2 mutations, as preceding investigations already revealed effectiveness of this approach against trastuzumab-resistant breast cancer [10]. The destabilization and subsequent degradation of ErbB2 are achieved by displacing HSP90 that chaperones ErbB2 conformation with HSP70 that leads to ErbB2 ubiquitylation through recruiting the ubiquitin ligase CHIP (C-terminal Hsc70-Interacting Protein), which process is enabled by various HSP90 inhibitors [11–13]. Accordingly, the anti-cancer effects of multiple HSP90 inhibitors have been experimentally demonstrated either as monotherapies or in combinations with other ErbB2-targeting agents [14, 15]. More importantly, initial clinical trials with the HSP90 inhibitors tanespimycin (17-AAG) and alvespimycin (17-DMAG), two derivatives of the antibiotic geldanamycin, provided additional lines of evidence that supports the validity of targeting HSP90 in ErbB2-positive breast cancers [16, 17].

Despite years of efforts to comprehend the HSP90 inhibitor-triggered ErbB2 downregulation, uncertainty still exists regarding the specifics of the itinerary of ubiquitylated ErbB2. It is also unfortunate that none HSP90 inhibitors fulfilled all requirements to become an FDA-approved therapy to treat ErbB2-positive breast cancer so far, partly owing to the adverse side effects associated with the inhibition of HSP90 that maintains the stability and functionality of a wide range of client proteins [18]. In the present study, we corroborate that HSP90 inhibition leads to the lysosomal degradation of ubiquitylated ErbB2, which process is counteracted by the deubiquitylase activity of Ubiquitin-Specific Protease 2 (USP2). Depletion and pharmacological suppression of USP2 effectively enhance HSP90 inhibitor-incurred ErbB2 downregulation, as well as significantly deter the in vivo and in vitro growth of ErbB2-positive breast cancer cells. Our findings provide novel insights into the dynamic regulation of HSP90 inhibitor-triggered ErbB2 downregulation and pave way for the development of alternative strategy to target ErbB2 via combined inhibition of HSP90 and USP2.

## Materials and methods

### Cell lines and transfection

Cell lines used in this study were obtained from the American Type Culture Collection (ATCC) and maintained at a humidified atmosphere in the CO_2_ incubator (Thermo, 3111) at 37 °C. Full growth medium was prepared by supplementing fetal bovine serum (Gibco, final concentration 10%) and penicillin/streptomycin (Thermo Fisher) into base medium. Specifically, ErbB2-positive breast cancer cell lines AU565, HCC1954, HCC1419, and ZR-75-30 were cultured with RPMI-1640 media, while SKBR3 was maintained using McCoy’s 5A media. HeLa, 4T1 and HEK293T cell lines were cultured with DMEM (Dulbecco’s Modified Eagle’s Medium) media. Transfection of plasmids into cells for immunofluorescence was performed using Lipofectamine 3000 (Invitrogen) according to the manufacturer’s instructions.

### Antibodies and other reagents

Mouse anti-ErbB2 (clones A-2 and 9G6) antibodies were purchased from Santa Cruz Biotechnology (CA, USA). Goat anti-ErbB2 (AF1129) antibody was obtained from R&D systems. Rabbit anti-USP2 antibody was purchased from Abgent. Mouse anti-GAPDH and anti-β-Actin antibodies were purchased from Proteintech (Wuhan, China). Mouse anti-tubulin antibody was purchased from Sigma. Rabbit anti-EEA1 antibody was purchased from Santa Cruz, and mouse anti-LAMP1 antibody was obtained from BD Medical Technology. Rabbit anti-HER2/ErbB2 (29D8) antibody was purchased from Cell Signaling Technology. Mouse anti-Ubiquitin (P4G7) antibody was purchased from Covance. Secondary (Infrared-labeled) goat anti-mouse and anti-rabbit, and donkey anti-goat antibodies were obtained from LI-COR. Chloroquine and cycloheximide were purchased from Sigma. HSP90 inhibitors 17-AAG, ganetespib, and PU-H71 were purchased from Dalian Meilun Biotechnology Co., Ltd (Dalian, China). ML364 was purchased from Medchem Express.

### Immunoblotting

Cells were lysed in ice-cold RIPA buffer (10 mM Tris-HCl pH 7.5, 150 mM NaCl, 1% Triton X-100, 0.1% SDS, and 1% sodium deoxycholate) supplemented with Na_3_VO_4_ (1 mM) and phenylmethylsulfonyl fluoride (PMSF, 1 mM). Protein concentrations were measured with BCA protein assay kit (Takara). Protein samples were separated on SDS-PAGE gels before transferred to nitrocellulose membranes (Merck Millipore, USA). Membranes were blocked for 2 h at room temperature in 4% fat-free milk in phosphate-buffered saline (PBS). Primary antibody incubation was carried out at cold room for overnight. The following day, blots were washed with PBS supplemented with 0.1% Tween 20 (PBST) for three times, prior to secondary antibody incubation at room temperature for 1 h. Following PBS washes, membranes were scanned on a LI-COR Odyssey imager according to the manufacturer’s instructions. Acquired data were quantitated using Image Studio software (Version 4.0).

### Immunoprecipitation and co-immunoprecipitation

In immunoprecipitation assays, cell lysates were prepared as abovementioned for immunoblotting. One milligram of lysate per condition was incubated with protein G-agarose and anti-ErbB2 antibody (9G6) for 4 h at 4 °C. Then agarose beads were washed three times with YP-IP buffer (0.1% Nonidet P-40, 25 mM Tris-HCl pH 7.5, 150 mM NaCl), before protein elution using 1.5× SDS-PAGE sample buffer. For immunoprecipitation under denatured conditions, cells were lysed in SDS lysis buffer (2% SDS, 1 mM EDTA, 50 mM NaF) preheated at 110 °C. Following protein concentration measurement, samples were diluted using four volumes of dilution buffer (12.5 mM Tris pH 7.5, 2.5% Triton X-100, 187.5 mM NaCl) before centrifugation to clear debris. Diluted lysates were then incubated with protein G-agarose and anti-ErbB2 antibody (9G6) for overnight at 4 °C. Immunoprecipitates were then washed three times with SDS wash buffer (10 mM Tris pH 7.5, 2% Triton X-100, 0.4% SDS, 150 mM NaCl) and eluted with 1.5× SDS-PAGE sample buffer. Samples were analyzed by immunoblotting with anti-ErbB2 and anti-ubiquitin antibodies. In co-immunoprecipitation assays, SKBR3 cells were treated with 500 nM of 17-AAG for indicated times before lysis with NP40 buffer (10 mM Tris-HCl pH 7.5, 150 mM NaCl, 0.5% Nonidet P-40). Cell lysates (1 mg per sample) were incubated with protein G-agarose and anti-ErbB2 antibody at 4 °C for 4 h. Following washes with YP-IP buffer, eluted protein samples were subjected to immunoblotting analyses with anti-ErbB2 and anti-USP2 antibodies.

### Cell cycle

Following indicated treatments, cells were harvested by trypsinization and counted, with half million per condition used in cell cycle analysis. Cells were PBS washed twice and fixed in ice-cold 70% ethanol for overnight, prior to incubation with 50 μg/ml of propidium iodide in PBS supplemented with 100 µg/ml of RNase at room temperature for 1 h. Samples were then processed using a bench-top flow cytometer (Accuri C6, BD Biosciences), with acquired data analyzed using FlowJo software version 7.6.1 (FlowJo, LLC, USA).

### Immunofluorescence

ErbB2-positive breast cancer cells were cultured on glass coverslips in 35-mm dish. Immunofluorescence assays were performed as described previously [19]. Briefly, cells were initially washed with PBS and fixed with 3% paraformaldehyde, before permeabilized with 0.2% Triton X-100. Samples were blocked in 10% goat serum for 1 h, and then incubated with primary antibodies for 1 h at room temperature. Subsequently, cells were stained with fluorescent secondary antibodies (Invitrogen, USA) at room temperature in the dark for 30 min. Then coverslips were washed sequentially in PBS and double distilled water, before mounted onto slides using Mowiol supplemented with DAPI to stain nucleus. Finally cells were examined under a fluorescent microscope (Leica, Germany). In confocal microscopy analysis, coverslips were treated same way as abovementioned. To examine co-localization of proteins of interest (ErbB2, EEA1, LAMP1), cells were incubated with two selected primary antibodies from different species, followed by secondary antibody incubation (Alexa Fluor^®^ 488 and 594-labeled, Invitrogen). Coverslips were mounted, and staining was examined under a laser scanning confocal microscope (Leica, TCS SP5).

### Colony formation assay

Cultured AU565, AU565-USP2-del, and AU565-USP2-del-CCND1 cells were trypsinized and counted, with 2000 cells seeded into 6-cm dish per condition. Plates were kept in the CO_2_ incubator at 37 °C, with growth medium replenished every other day in a duration of 2 weeks. Similarly, SKBR3 and HCC1954 cells were harvested and plated into 35-mm dishes at a seeding density of 1000 cells per plate, which were maintained in the CO_2_ incubator for 1 week. Cells were then treated with 17-AAG (100 nM) and ML364 (10 μM), with inhibitors refreshed every 24 h for a duration of 1 week. Cell colonies were fixed with methanol and then stained with 0.1% crystal violet. Plates were imaged using a Bio-Rad ChemiDoc XRS+ system, and acquired data were analyzed using ImageJ program.

### Stable cell lines

To generate SKBR3 cells with stable knockdown of selected deubiquitylases (DUBs), target-specific shRNA vectors (pLKO.1) were obtained from Sigma and used to transfect HEK293T cells together with lentivirus packaging plasmids according to the manufacturer’s instructions. Collected viruses were used to infect SKBR3 cells and positive cells were selected with puromycin incubation at 2 μg/ml. Two shRNA sequences were used per target, with pLKO.1 empty vector included as negative control. The target sequences of the shRNA clones used in this study are described in Supplementary Table 1. The knockdown efficiency of selected targets was validated by RT-PCR, and stable cell lines were routinely maintained in culture media supplemented with 1 μg/ml of puromycin throughout all experiments.

To generate USP2 knockout cell line, CRISPR/Cas9-based genome editing protocol was followed [20]. Briefly, guide RNAs were designed with the online tool (http://crispr.mit.edu/) and the target sequences are as follows: sgRNA#1A, ACCTAGAATCCTTTCATAGGAGG; and sgRNA#1B, CCTCTGGTAGTAAGTAACACAGG. Oligonucleotides were commercially synthesized with overhangs to enable ligation into expression vectors. To allow homologous recombination, G418 resistance cassette was flanked with USP2 sequences from PCR amplification using the following primer sets: USP2-5′-SacII-F, GCCCGCGGCAGGAGTGCTTCTGTAGACAGA; USP2-5′-NotI-R, GCGCGGCCGCTGAAAGGGGTGAAGTGAGGCAA; USP2-3′-SalI-F, GCGTCGACCCTCTGTCATGTGCCCCTG, and USP2-3′-EcoRV-R, GCGATATCCTGTCCCTGGACTGCGACA. Following construct verification by sequencing, AU565 cells were co-transfected with guide RNA and Cas9/D10A expression vectors, along with linearized USP2 sequence-sandwiched G418 resistance cassette. After 48 h of transfection, cells were treated with 800 μg/ml of G418 for 1 week, before single cells sorted into each well of 96-well plates using a cell sorter (BD, FACSAria II). Cell clones were expanded in the presence of G418, with USP2 expression examined by immunoblotting to identify positive knockout cells.

To complement cyclin D1 expression, *CCND1* sequence was cloned into pCDH expression vector, using which lentiviruses were prepared via co-transfection into HEK293T cells together with psPAX2 and pMD plasmids. AU565-USP2-Del cells generated by CRISPR/Cas9 technology were infected with pCDH-CCND1 lentiviruses and positive expression cells were screened with puromycin at 2 μg/ml. Cyclin D1 expression in established AU565-USP2del-CCND1 cells was finally validated by immunoblotting, and stable cells were maintained in G418 (800 μg/ml) and puromycin (1 μg/ml) containing media throughout all experiments. Similarly, *ErbB2* sequence was subcloned into pCDH plasmid and used for lentivirus packing to generate 4T1 cells with stable expression of ErbB2.

### Xenograft mouse model

Experimental procedures regarding the animal study have been approved by the Institutional Animal Care and Use Committee at Dalian Medical University. To generate breast cancer xenografts, ErbB2-overexpressing HCC1954 cells (2 million/mouse) were subcutaneously inoculated into female nude mice (4–6–week–old Balb/c background, Vital River Laboratories, Beijing). Following inoculation, mice were housed under specific pathogen-free conditions throughout the experiments, with xenograft sizes measured using a vernier caliper every other day. After the average size of xenograft tumors reached about 160 mm^3^, mice were randomly divided into four groups (5 mice per group), with each group having approximately equal mean tumor volumes and administered with vehicle, ML364, 17-AAG, or the combination of ML364 and 17-AAG. ML364 and 17-AAG were both administered by intraperitoneal injection, with ML364 at daily dosage of 5 mg/kg and 17-AAG every two days at 50 mg/kg during a 3-week duration. Tumor volumes were calculated by measuring the length (*L*) and width (W) with calipers using the formula: volume = (1/2) × *L* × *W*^2^. Same procedures were followed for the syngeneic xenograft mouse model, after ErbB2-expressing 4T1 cells were implanted subcutaneously into normal female mice with Balb/c background (1 million/mouse).

### Immunohistochemistry

Resected xenograft tumor tissues were fixed with 4% paraformaldehyde before embedded in paraffin. Prepared tissue sections (4 μm) were deparaffinized with xylene and rehydrated with graded alcohol. Tissue sections were then treated with blocking solution (ZSGB-Bio) for 30 min at room temperature, before primary antibody incubation for overnight at 4 °C, followed by secondary antibody incubation for 1 h at room temperature. Tissue samples were stained using a peroxidase IHC assay kit (ZSGB-Bio, Beijing) according to the manufacturer’s instructions. Mounted sections were examined by light microscopy (Leica) and acquired images were analyzed with Image-Pro Plus software (version 6.0).

### Statistics

Biologically repeated experiments (*n* ≥ 3) were carried out to allow statistical analysis. Results were generally illustrated by mean ± standard error of the mean (SE). Two-tailed Student’s *t* test was performed in GraphPad Prism software (version 7) to evaluate statistical differences between groups, with calculated *p* values of less than 0.05 deemed as significant differences.

## Results

### HSP90 inhibition leads to the internalization and ubiquitylation-mediated degradation of ErbB2

**Fig. 1:**
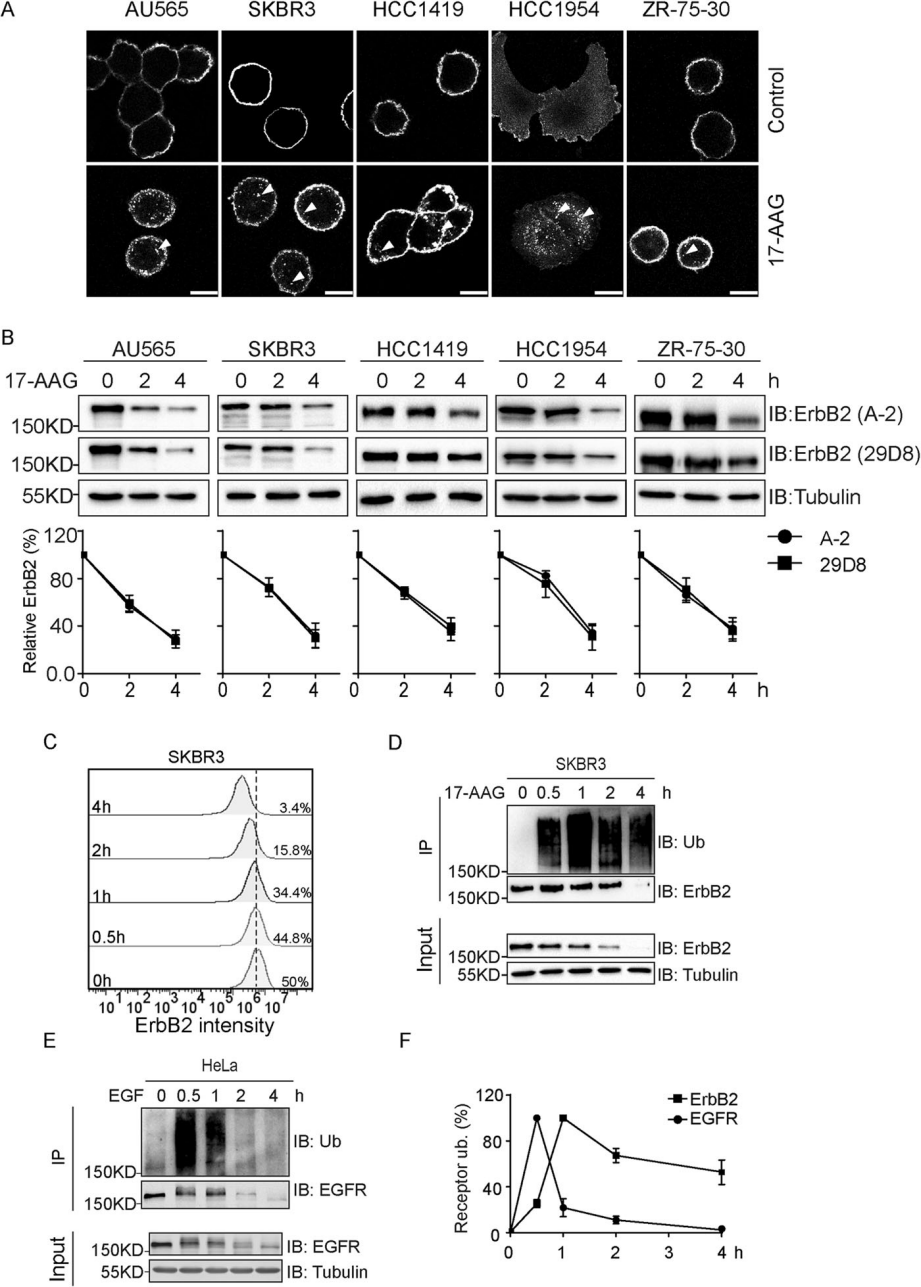
HSP90 inhibitor-incurred ErbB2 ubiquitylation leads to its internalization and degradation. **a** ErbB2-positive breast cancer cells were treated with the HSP90 inhibitor 17-AAG (500 nM) for 4 h or left untreated (control) before immunofluorescence assays to examine ErbB2 localization. Images show representative confocal sections, with triangles pointing to intracellular ErbB2 punctae. Scale bar = 10 μm. **b** Cultured ErbB2-positive breast cancer cells were subjected to 17-AAG treatment at 500 nM for indicated times and lysed for immunoblotting using two clones of anti-ErbB2 antibodies. Tubulin was probed to show equal loading. Degradation curves show correlating quantification of relative ErbB2 intensities (0 h set as 100%). **c** SKBR3 cells were treated with 500 nM of 17-AAG for indicated times and harvested for flow cytometric analysis to examine surface levels of ErbB2. **d** SKBR3 cells were treated with 500 nM of 17-AAG for indicated times and lysed. ErbB2 proteins were immunoprecipitated using mouse anti-ErbB2 antibody (clone 9G6) and analyzed by immunoblotting with anti-ubiquitin antibody, with immunoblotting analysis of cell lysates performed in parallel to examine cellular levels of ErbB2 and Tubulin. **e** Cultured HeLa cells were treated with 20 ng/ml of EGF for indicated times and lysed. EGFR was immunoprecipitated from each sample and analyzed by immunoblotting with ubiquitin antibody to examine the ubiquitylation of EGFR. Cell lysates were also analyzed by immunoblotting to probe for EGFR and Tubulin. **f** Relative quantification data of ubiquitin signal after ErbB2 and EGFR normalization from (**d**) and (**e**), respectively. The samples showing highest intensities were considered as 100%, with 0.5 h and 1 h set for EGFR and ErbB2, respectively. All error bars represent standard error of the mean (*n* = 3).

Since the observation of geldanamycin-incurred ErbB2 degradation, different intracellular itineraries and degradation pathways for ubiquitylated ErbB2 have been proposed in ErbB2-positive breast cancer cells exposed to HSP90 inhibitors [11, 21–24]. To interrogate the whereabouts of ErbB2 following HSP90 inhibition, we carried out immunofluorescence assays to investigate the intracellular distribution of ErbB2 in a panel of ErbB2-overexpressing breast cancer cells (AU565, SKBR3, HCC1419, HCC1954, and ZR-75-30) treated with 17-AAG, a widely used HSP90 inhibitor with clinical efficacy [16]. As demonstrated in Fig. 1a, 17-AAG treatment dramatically enhanced the internalization of ErbB2, also in HCC1954 cells that already showed cytoplasmic ErbB2 staining without treatment. As expected, ErbB2 expression was downregulated in all breast cancer cell lines tested as revealed by immunoblotting assays using two clones of ErbB2 antibodies (Fig. 1b).

In keeping with immunoblotting data, results from flow cytometric analysis also reveal a gradual decrease in the cell surface presence of ErbB2 from SKBR3 cells treated with 17-AAG (Fig. 1c). Having demonstrated that both immunoblotting and flow cytometry assays showed consistent ErbB2 downregulation following 17-AAG addition, we turned to inspect the ubiquitylation status of ErbB2 receptor in SKBR3 cells exposed to 17-AAG. As illustrated in Fig. 1d, in accordance with results from previous studies, 17-AAG treatment effectively induced the ubiquitylation of ErbB2 in SKBR3 cells. However, when compared with the EGF-induced EGFR ubiquitylation that was abrupt and transient, ubiquitylated ErbB2 incurred by 17-AAG accumulated gradually and appeared more durable (Fig. 1d–f). Furthermore, to examine whether ubiquitylation occurred on ErbB2 directly rather than on associated interactors, we carried out ErbB2 immunoprecipitation under denatured conditions to remove its interacting partners, from which we confirmed direct ubiquitylation of ErbB2 following 17-AAG treatment (Supplementary Fig. 1a–c).

### 17-AAG triggers the endo-lysosomal sorting of ErbB2

**Fig. 2:**
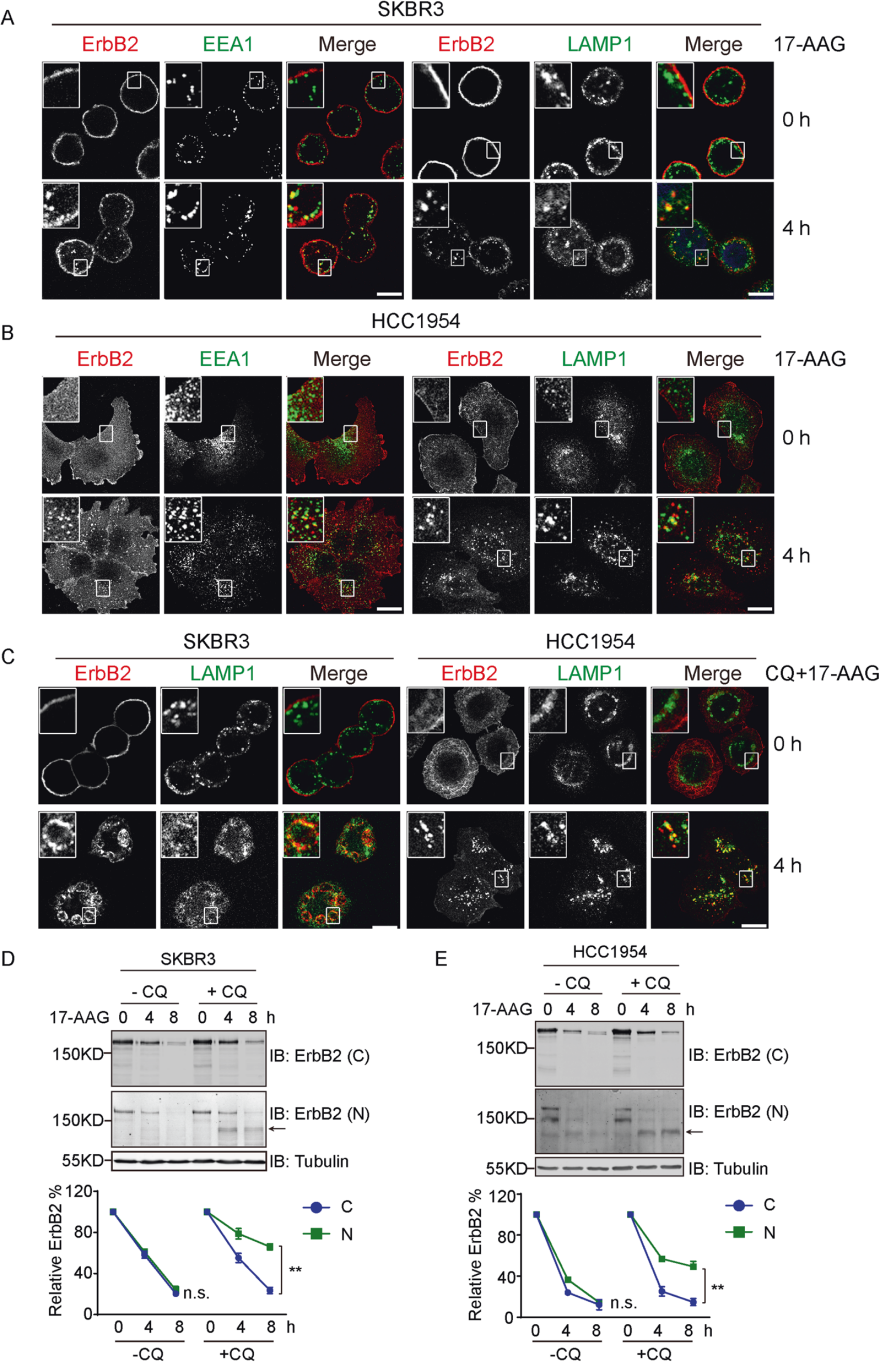
17-AAG induces lysosomal degradation of ErbB2 through endo-lysosomal trafficking. **a** **b** SKBR3 and HCC1954 cells were treated with 17-AAG at 500 nM for 4 h, before processed for immunofluorescence assays along with untreated control cells. Cells were labeled with anti-ErbB2 antibody together with either anti-EEA1 or anti-LAMP1 antibodies as indicated. Representative confocal sections are shown with magnified insets to illustrate colocalizations of ErbB2 with EEA1 and LAMP1. Scale bar = 10 μm. **c** SKBR3 and HCC1954 cells were pretreated with 100 μM of chloroquine (CQ) for 30 min before exposed to 17-AAG for 4 h (0 h as untreated controls). Samples were then analyzed by immunofluorescence and confocal microscopy using anti-ErbB2 and anti-LAMP1 antibodies. Micrographs show representative confocal sections, with magnified insets demonstrating colocalizations. Scale bar = 10 μm. **d**, **e** SKBR3 and HCC1954 cells were pretreated with chloroquine at 100 μM for 30 min (DMSO as control) prior to 17-AAG addition for indicated times. Cell lysates were analyzed by immunoblotting with antibodies directed against C- and N-termini of ErbB2. Tubulin was probed to show equal loading. Degradation curves show relative quantification of ErbB2 intensities (including truncated version) in corresponding samples. Error bars represent standard error of the mean (*n* = 3), with n.s. not significant and **p < 0.01.

The EGF-induced endocytic degradation of EGFR has been extensively studied and represents a classical paradigm of RTK endocytosis [25, 26]. Interestingly, our observations of 17-AAG induced ErbB2 downregulation from 5 ErbB2-positive breast cancer cell lines imply similar molecular features much akin to the endocytic degradation of EGFR. To this end, we carried out confocal microscopy to examine the colocalizations of internalized ErbB2 with early endosomes and late endosomes/lysosomes using their specific markers EEA1 and LAMP1, respectively. Results from confocal analyses demonstrate apparent distribution of internalized ErbB2 to compartments with positive staining for EEA1 or LAMP1 in SKBR3, HCC1954, AU565, HCC1419, and ZR-75-30 cells treated with 17-AAG, indicating ErbB2 traveling to endo-lysosomal routes post-HSP90 inhibition (Fig. 2a, b, Supplementary Fig. 2).

To assess the involvements of lysosome function in 17-AAG-incurred ErbB2 degradation, we treated ErbB2-positive breast cancer cells with chloroquine that blocked the acidification of lysosomes. Under such circumstances, internalized ErbB2 was observed to accrue on LAMP1-positive structures that represented abnormally enlarged lysosomes (Fig. 2c and Supplementary Fig. 3). In addition, immunoblotting results showed that chloroquine treatment recovered a truncated species of ErbB2 that was recognized by antibody raised against N-terminus but not C-terminus of ErbB2, in both SKBR3 and HCC1954 cells treated with 17-AAG, suggesting that ErbB2 routed to the lysosomes was subjected to proteolytic cleavage from the C-terminal part prior to lysosomal degradation (Fig. 2d, e).

### USP2 counteracts the endocytic degradation of ErbB2 through deubiquitylation

**Fig. 3:**
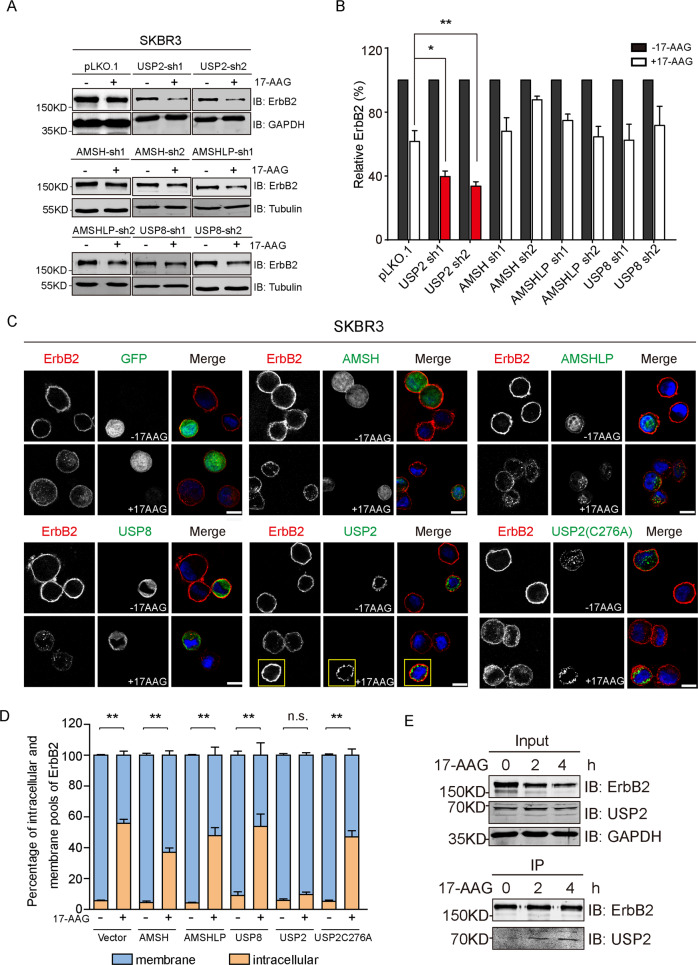
USP2 precludes endocytic degradation of ErbB2 triggered by HSP90 inhibition in a DUB activity-dependent manner. **a** SKBR3 cell lines with stable knockdown of USP2, AMSH, AMSHLP, and USP8 (two shRNAs per target and empty vector pLKO.1 as control) were treated with 80 nM of 17-AAG or DMSO for 10 h. Cell lysates were subjected to immunoblotting analysis using anti-ErbB2 antibody. GAPDH or Tubulin was probed to confirm equal loading. **b** quantification data of relative ErbB2 intensities from (**a**). **c** SKBR3 cells were transfected with constructs expressing GFP, GFP-AMSH, GFP-AMSHLP, GFP-USP8, GFP-USP2, and GFP-USP2(C276A) as indicated. Cells were treated with DMSO or 17-AAG (500 nM) for 4 h and then processed for immunofluorescence analysis using anti-ErbB2 antibody. Nucleus was stained with DAPI. Images show representative confocal sections. GFP-USP2 expressing cell was indicated with solid box. Scale bar = 10 μm. **d** Intensities of the membrane and intracellular pools of ErbB2 were quantified using results from C with Image Studio software (Version 4.0) and plotted. **e** SKBR3 cells were treated with 500 nM of 17-AAG for indicated times and lysed. ErbB2 was immunoprecipitated from cell lysates using mouse anti-ErbB2 antibody (clone 9G6). Cell lysates (input) and immunoprecipitation (IP) samples were analyzed by immunoblotting with indicated antibodies. Error bars represent standard error of the mean (*n* = 3), with n.s. not significant, *p < 0.05, and **p < 0.01.

The posttranslational ubiquitylation of substrate protein is a dynamic process that can be reversed by DUBs and several DUB activities have been implicated in the regulation of receptor endocytosis, which prompts us to speculate the involvement of DUB activity during the HSP90 inhibitor-incurred ErbB2 downregulation [27]. In doing this, we screened a panel of 15 DUBs that showed cytoplasmic distribution including four DUBs (AMSH, AMSHLP, USP2, and USP8) that were previously reported to locate to the endo-lysosome system, for their ability to influence 17-AAG-induced ErbB2 downregulation [28]. Each candidate DUB was targeted by two specific shRNAs and a panel of 30 DUB-targeting shRNA-expressing stable SKBR3 cell lines was established in addition to empty vector control cells. We first examined ErbB2 levels in these cells cultured under normal conditions and observed significantly reduced ErbB2 abundance in both USP2-depleted cell lines (Supplementary Fig. 4a, b). Furthermore, we treated this panel of DUB knockdown cell lines with 17-AAG and examined the influence of DUB silencing on the downregulation of ErbB2, with quantification data from immunoblotting analysis showing that the depletion of USP2 significantly enhanced the downregulation of ErbB2 incurred by 17-AAG (Supplementary Fig. 4c, d). Consistently, in another round of screening focusing on the 4 DUBs associated with endocytic pathway in which all four candidates were effectively silenced, only USP2 knockdown significantly enhanced the 17-AAG-incurred downregulation of ErbB2 (Fig. 3a, b; Supplementary Fig. 5a).

To validate the negative regulation that USP2 displayed toward 17-AAG-induced downregulation of ErbB2, we turned to an alternative approach to investigate the influence of DUB overexpression through immunofluorescence assays. As demonstrated in Fig. 3c, d, the transient overexpression of GFP and GFP-tagged DUBs (AMSH, AMSHLP, USP8, USP2, and the catalytic inactive USP2-C276A) did not significantly affect the expression levels and subcellular distribution of ErbB2 in SKBR3 cells under steady state conditions. In accordance with previous observations, 17-AAG treatment effectively triggered the internalization of ErbB2 with concomitantly reduced cell membrane distribution. This process was nevertheless precluded in cells transfected with wild-type USP2 but not the catalytic inactive mutant C276A and any other DUBs as revealed by immunofluorescence assay and quantification data of membrane and intracellular pools of ErbB2 (Fig. 3c, d). Therefore, our findings from knockdown and overexpression experiments consistently indicate that USP2 functions as a negative regulator of 17-AAG-induced ErbB2 degradation in a DUB activity-dependent manner. Additionally, in the subsequent co-immunoprecipitation analysis to examine the association of USP2 with ErbB2, USP2 was readily pulled down by immunoprecipitated ErbB2 especially in cells treated with 17-AAG, suggesting stronger association between USP2 and ubiquitylated ErbB2 (Fig. 3e).

### Pharmacological inhibition of USP2 destabilizes ErbB2 and potentiates HSP90 inhibition-incurred ErbB2 downregulation

**Fig. 4:**
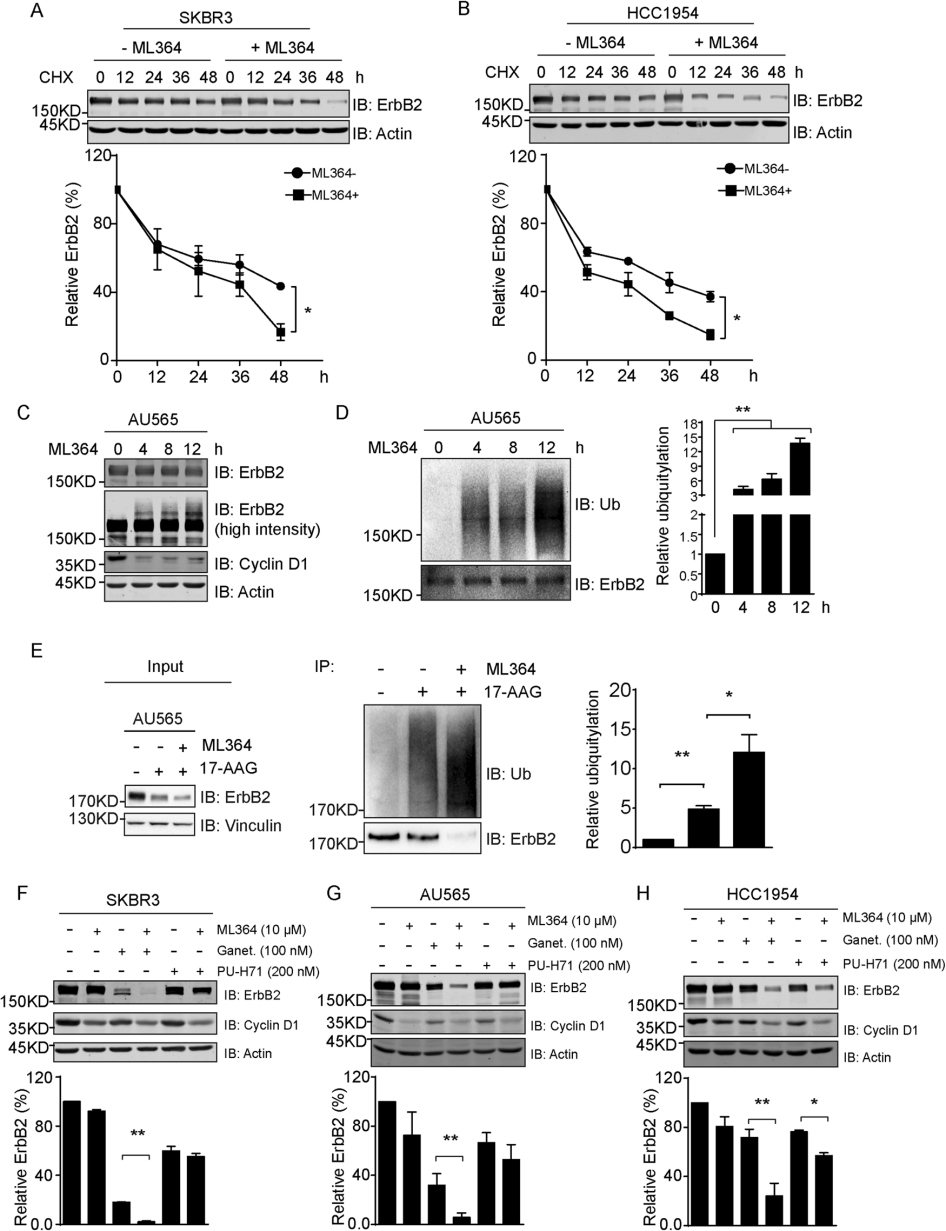
The USP2 inhibitor ML364 accelerates ErbB2 turnover via inducing its ubiquitylation and potentiates HSP90 inhibitors to degrade ErbB2. **a**, **b** SKBR3 and HCC1954 cells were treated with cycloheximide (100 μg/ml) in the absence or presence of 10 μM of ML364 for indicated times and lysed. Samples were analyzed by immunoblotting with indicated antibodies. Actin blots show equal loading. Turnover curves show relative quantification of ErbB2 expression at corresponding time points. **c** AU565 cells were treated with 10 μM of ML364 for indicated times. Cell lysates were prepared and analyzed by immunoblotting using indicated antibodies. Actin was probed to show equal loading. **d** AU565 cells were treated as in (**c**), and ErbB2 was immunoprecipitated using mouse anti-ErbB2 antibody (clone 9G6) from cell lysates, before immunoblotting assays to probe for ubiquitin and ErbB2. Column chart on the right shows the quantification of relative ubiquitin signal after ErbB2 normalization. **e** AU565 cells were treated with 17-AAG at 80 nM in the absence or presence of 10 μM of ML364 as indicated for 12 h. ErbB2 was immunoprecipitated using mouse anti-ErbB2 antibody (clone 9G6) from cell lysates and analyzed by immunoblotting to examine the ubiquitylation status of ErbB2 along with cell lysates. Column chart on the right shows the relative quantification of ubiquitin signal detected from immunoprecipitations after ErbB2 normalization. **f**–**h** SKBR3, AU565, and HCC1954 cells were treated with ML364, ganetespib, PU-H71, or various combinations at indicated concentrations for 12 h and lysed. Cell lysates were subjected to immunoblotting analysis with indicated antibodies. Actin blots are shown to confirm equal loading. The column charts show corresponding quantification of relative ErbB2 levels. Error bars represent standard error of the mean (*n* = 3), with *p < 0.05 and **p < 0.01.

Taking advantage of the successful development of the USP2 inhibitor ML364, we investigated the influence of pharmacological USP2 inhibition on ErbB2 expression in breast cancer cells [29]. In a series of cycloheximide chase experiments to examine the stability of ErbB2 in SKBR3 and HCC1954 cells with ML364 treatment, ErbB2 appeared to be consistently turned over more rapidly in the presence of this USP2 inhibitor relative to control samples and cells treated with the USP14 inhibitor IU1 or the USP7 inhibitor P5091 (Fig. 4a, b; Supplementary Fig. 6a, b) [30, 31]. It is noteworthy that we observed increasingly stronger smear signal of ErbB2 with immunoblotting analysis using an increasing duration of ML364 treatment, which indicated gradually enhanced ubiquitylation of ErbB2 (Fig. 4c). Indeed, such increase in ErbB2 ubiquitylation was further confirmed with immunoprecipitated ErbB2 and appeared to be dramatically stronger in ML364-treated cells than in those treated with IU1 or P5091 (Fig. 4d; Supplementary Fig. 6c). In this assay, we also detected cyclin D1 expression, given that cyclin D1 was identified as a bona fide substrate for USP2 [32]. Our data show consistently decreased cyclin D1 levels in ML364-treated samples, suggesting efficient suppression of USP2 function by ML364 (Fig. 4c). Therefore, it seems that the USP2 regulation on ErbB2 levels is not restricted to scenarios of HSP90 inhibition, but ErbB2 is constantly under the protection of the DUB activity from USP2 to avoid ubiquitylation and subsequent degradation.

We then investigated the impact of ML364 on 17-AAG-induced ErbB2 downregulation. Consistent with results from USP2 knockdown analysis, the addition of ML364 resulted in further decreased ErbB2 levels in AU565 cells treated with 17-AAG, accompanied with dramatically elevated ErbB2 ubiquitylation comparing to samples treated with 17-AAG alone or in the presence of IU1 or P5091 (Fig. 4e; Supplementary Fig. 6d). Given that different classes of HSP90 inhibitors have been developed till now, which include the resorcinol-based and purine-like HSP90 inhibitors in addition to the ansamycin-based inhibitors that were represented by geldanamycin and 17-AAG, we next evaluated the combined effects of ML364 with ganetespib (STA-9090) and PU-H71 that were from resorcinol-based and purine-like types, respectively [33]. As demonstrated in Fig. 4f–h, both ganetespib and PU-H71 led to efficient downregulation of ErbB2 in SKBR3, AU565, and HCC1954 cells, although to different extent, while the combined treatment with ML364 elicited significantly stronger inhibition on ErbB2 expression. It hence implies that the USP2 inhibitor ML364 is capable of potentiating a wide range of HSP90 inhibitors to suppress ErbB2 levels in breast cancer cells.

### ML364 sensitizes breast cancer cells to HSP90 inhibition

**Fig. 5:**
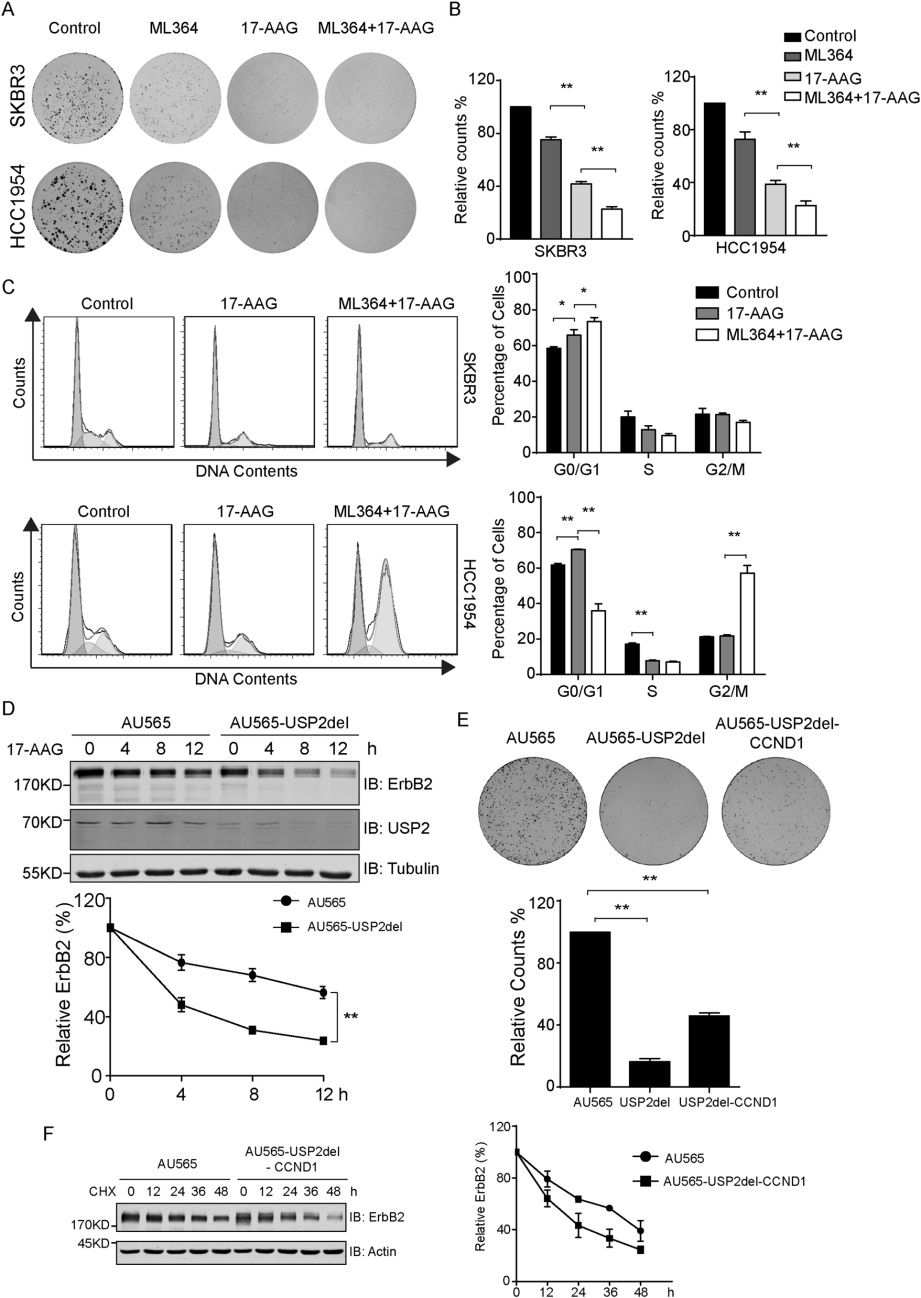
Pharmacological USP2 inhibition sensitizes ErbB2-positive breast cancer cells to HSP90 inhibition. **a** Colony formation assays carried out with SKBR3 and HCC1954 cells under the treatment of vehicle, ML364 (10 μM), 17-AAG (100 nM), and the combination of ML364 (10 μM) and 17-AAG (100 nM). **b** Quantification data from **a** showing the relative amounts of colonies formed in each group. **c** SKBR3 and HCC1954 cells were treated as in **a** for 24 h and then processed for cell cycle analysis. Representative histograms are shown to illustrate cell cycle distributions. Column charts on the right demonstrate quantification data of cells within each stage as indicated. **d** AU565 and AU565-USP2del cells were treated with 80 nM of 17-AAG for indicated times. Cell lysates were subjected to immunoblotting analysis using indicated antibodies. Degradation curves show the relative quantification of ErbB2 levels at corresponding time points. **e** Colony formation assays performed using AU565, AU565-USP2del, and AU565-USP2del-CCND1 cells. Column chart shows the relative amounts of colonies formed in each cell line. **f** AU565 and AU565-USP2del-CCND1 cells were treated with 100 μg/ml of cycloheximide for indicated times and lysed. Samples were analyzed by immunoblotting using indicated antibodies. Actin was probed to confirm equal loading. The turnover curve on the right shows the quantification of relative ErbB2 abundance from immunoblotting assays. Error bars represent standard error of the mean (*n* = 3), with *p < 0.05 and **p < 0.01.

Through chaperoning the active conformations of a plethora of clients including diverse kinases and transcription factors, HSP90 functions as a major contributor to the maintenance of cellular proteostasis [34]. It is hence conceivable that the disruption of HSP90 functionality will lead to lethal damages to a wide range of cell types from both cancer and normal tissues. Such pleiotropic effects of HSP90 inhibition render a certain HSP90 inhibitor a corresponding therapeutic window in its clinical applications. Having observed the potentiating effects of USP2 inhibition toward 17-AAG-induced ErbB2 downregulation, we reasoned that the USP2 inhibitor ML364 might sensitize ErbB2-positive breast cancer cells to HSP90 inhibition and accordingly investigated the effectiveness of the combined treatment. As described in Fig. 5a–c, results from colony formation and cell cycle analyses revealed that ML364 significantly enhanced the inhibitory effects of 17-AAG to hinder the cell cycle progression and curb the growth of breast cancer cells with ErbB2 overexpression.

It is worth noting that cyclin D1 has been established as a USP2 substrate, and ML364 has been shown to induce cell cycle arrest via downregulating cyclin D1 [29, 32]. In accordance with these observations, we consistently detected decreased levels of cyclin D1 in ML364-treated cells and in an AU565 stable cell line in which we successfully knocked out one *USP2* allele using CRISPR/Cas9 technology and observed enhanced ErbB2 downregulation following 17-AAG treatment (Fig. 4c, f–h, d and Supplementary Fig. 5 b-complete knockout of USP2 was not achieved likely due to severe growth defect). To remove the influence of growth defect attributed by cyclin D1 downregulation, we restored cyclin D1 expression in this USP2 half-knockout stable cell line (AU565-USP2del) and generated another stable cell line with cyclin D1 overexpression (AU565-USP2del-CCND1). Compared with parental AU565 cells, the turnover of ErbB2 was consistently accelerated in AU565-USP2del-CCND1 (Fig. 5f). Furthermore, although the complementation of cyclin D1 partially rescued the growth defect observed in AU565-USP2del cells, the capacity of AU565-USP2del-CCND1 to form colonies was substantially compromised comparing to parental AU565 cells (Fig. 5e). These findings collectively suggest that, apart from resulting in cell cycle arrest incurred by cyclin D1 reduction, USP2 inhibition can lead to declined propagation of ErbB2-postive breast cancer cells through interfering with ErbB2.

### Combination of USP2 and HSP90 inhibitors effectively restrains ErbB2-positive breast cancer xenograft growth

**Fig. 6:**
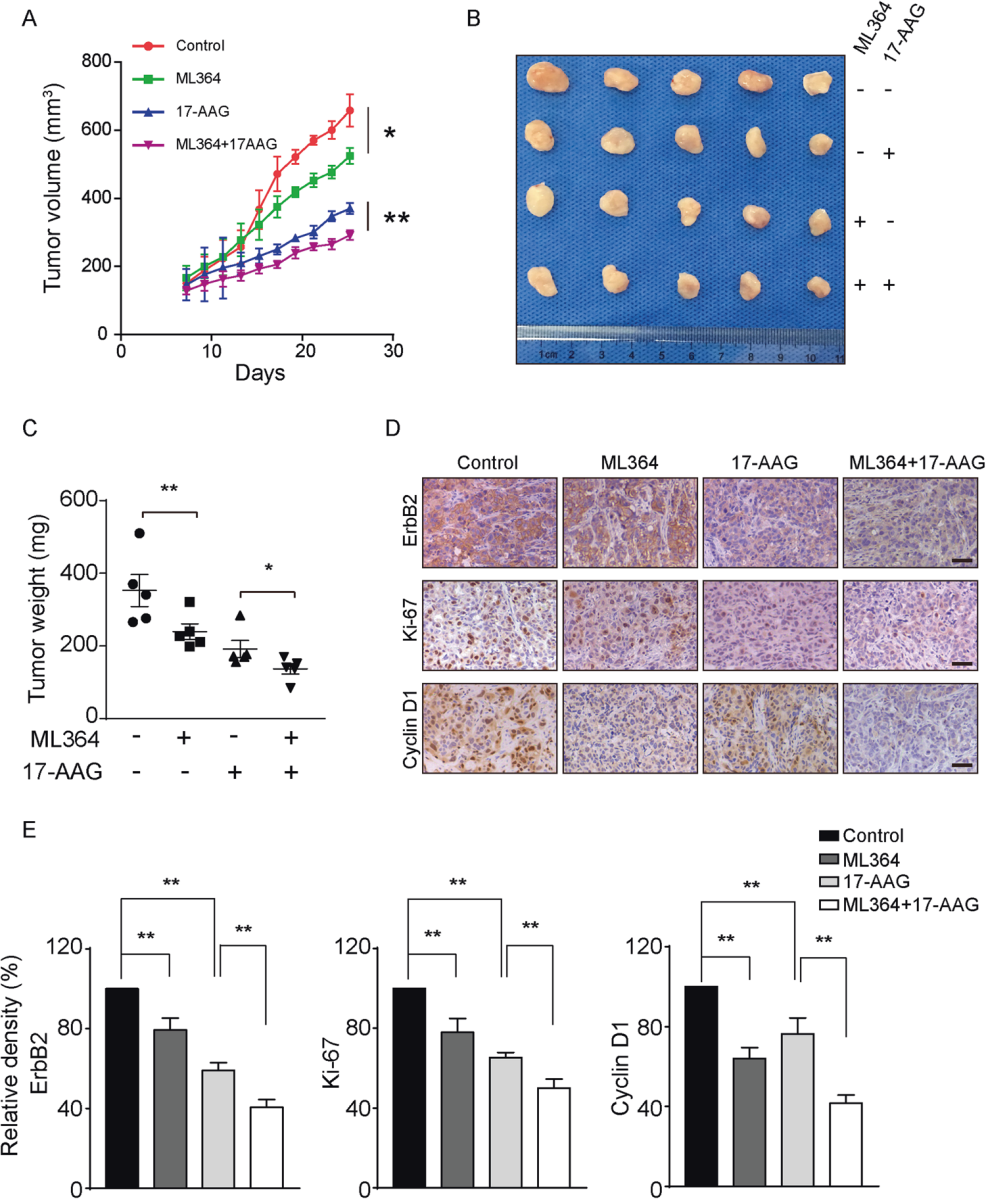
The combination of ML364 and 17-AAG effectively suppresses HCC1954 xenograft growth. **a** HCC1954 cells were inoculated into female nude mice to generate xenograft mouse models. Mice were randomized into four groups to receive control vehicle, ML364, 17-AAG, or the combination of ML364 and 17-AAG. The sizes of xenografts were measured every 2 days and calculated tumor volumes were plotted. **b** Resected xenograft tumor samples from each treatment group. **c** Tumor weights were measured and plotted. **d** Tumor tissue sections were analyzed by immunohistochemistry using anti-ErbB2, anti-Ki-67, and anti-cyclin D1 antibodies. Images show representative xenograft sections. Scale bar = 50 μm. **e** Column charts show quantification data (integral optical density) of relative intensities for ErbB2, Ki-67, and cyclin D1. Error bars represent standard error of the mean (*n* = 5), with *p < 0.05 and **p < 0.01.

**Fig. 7:**
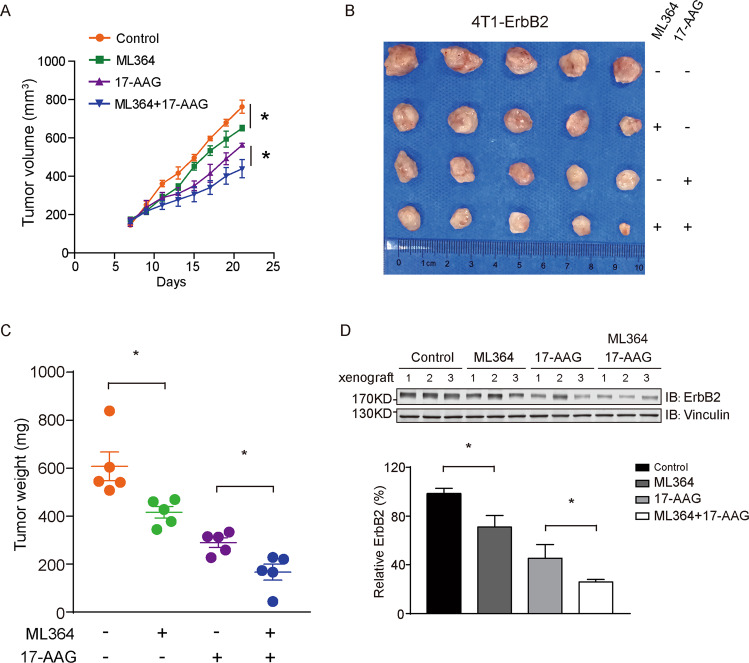
The combined treatment with ML364 and 17-AAG inhibits tumor growth in a syngeneic xenograft mouse model. **a** 4T1 cells stably expressing ErbB2 (4T1-ErbB2) were implanted into female Balb/c mice for xenograft formation. Mice were randomly divided into four groups and administered with control vehicle, ML364, 17-AAG, or the indicated combination. The xenograft volumes were measured and plotted. **b** Xenografts resected from mice receiving different treatment as indicated. **c** Tumor weights of xenograft samples from each treatment group. **d** Immunoblotting analysis of tumor tissue samples using indicated antibodies to detect ErbB2 levels. Vinculin blots show equal loading. Column chart shows relative quantification of ErbB2 expression. Error bars represent standard error of the mean (*n* = 5), with *p < 0.05.

**Fig. 8:**
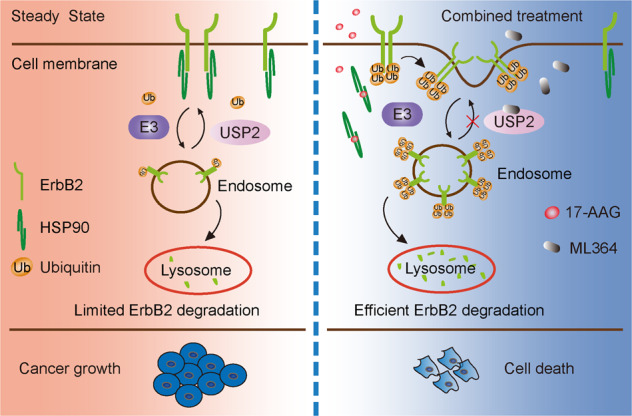
Schematic diagram depicting the influence of USP2 and HSP90 inhibitors on cell surface ErbB2. **a** The current working model shows the regulation of cell surface ErbB2 by USP2 and HSP90. Under steady state conditions, USP2 protects ErbB2 surface levels by restricting its ubiquitylation. Through inhibiting USP2, ML364 potentiates the pro-degradation effects of HSP90 inhibitors toward ErbB2, which collectively elicit potent anti-cancer effects.

Next we sought to evaluate the combination of USP2 and HSP90 inhibitors using ErbB2-positive breast cancer xenograft mouse models. In doing this, we subcutaneously implanted HCC1954 cells into athymic female nude mice. Following xenograft formation with average volumes of ~160 mm^3^, mice were randomly divided into four groups that were administered with control vehicle, ML364, 17-AAG, or the combination of ML364 and 17-AAG. As demonstrated in Fig. 6a–c, ML364 and 17-AAG monotherapies both effectively suppressed the growth of HCC1954 xenografts, with 17-AAG treatment eliciting a stronger inhibition. Importantly, the combinatorial scheme incorporating both USP2 and HSP90 inhibitors most potently repressed the further in vivo expansion of the HCC1954 breast cancer xenografts, which was evidenced by markedly attenuated tumor volumes and weights (Fig. 6a–c). In accordance with our data from in vitro investigations, results from immunohistochemical analysis of HCC1954 xenograft tissues revealed significantly reduced staining of ErbB2 in ML364, 17-AAG, and combo treatment groups, with concomitantly decreased expression of the proliferation marker Ki-67 and cyclin D1 (Fig. 6d, e). Furthermore, we validated the efficacy of the ML364 and 17-AAG co-treatment using a syngeneic xenograft mouse model. Immunocompetent female Balb/c mice were subcutaneously inoculated with 4T1 cells stably expressing ErbB2 and treated as described (Fig. 7a; Supplementary Fig. 5c). Consistent with results from HCC1954 xenograft study, the combined treatment with ML364 and 17-AAG displayed the strongest suppression against xenograft tumor growth, which was accompanied with potent ErbB2 downregulation (Fig. 7b–d). Taken together, our findings unveil USP2 as a guardian of ErbB2 abundance in ErbB2-positive breast cancers, with its DUB activity required to antagonize the endocytic degradation of ErbB2 under steady state conditions (Fig. 8). Further investigations provide several lines of evidence that USP2 inhibition effectively sensitizes ErbB2-positive breast cancer cells to HSP90 inhibitors through enhancing ErbB2 downregulation (Fig. 8)

## Discussion

Recent global cancer statistical analysis has revealed breast cancer as the most frequently diagnosed type of cancer and the foremost cause of cancer-related death among females, with over 2 million new cases and more than 0.6 million deaths estimated worldwide in 2018 [35]. ErbB2-positive subtype, together with the hormone receptor-positive/ErbB2-negative and triple-negative subtypes, represents the major molecular classification of breast cancer patients that guides the selection of therapeutic strategies in the clinical treatment [36]. Accordingly, ErbB2-targeted therapies have profoundly improved prognoses of patients with ErbB2 overexpression, proving the validity and feasibility of this approach in addition to warrant further investigations [37].

Receptor endocytosis is a major means employed by cells to regulate the homeodynamics of cell surface proteins [38, 39]. In clear contrast to its family member EGFR, ErbB2 is endocytosis-deficient under normal conditions, with multiple mechanisms proposed for its surface retention [40–42]. We have recently reported the influence of cell membrane fluidity and rigidity on the surface accumulation of ErbB2, which can be exploited to design therapeutic strategies against ErbB2-positive breast cancer [43]. It is thus likely that multiple factors function together to coordinate the ErbB2 localization, while HSP90 inhibition efficiently disrupts such regulation and leads to ErbB2 degradation. Using a panel of five breast cancer cell lines with ErbB2 overexpression, we explicitly demonstrated that HSP90 inhibitor induced the endocytosis and lysosomal degradation of ErbB2, which are accompanied by the ubiquitylation of ErbB2. As a countervailing measure, USP2 negatively regulates this process to mitigate ErbB2 degradation in a deubiquitylase activity-dependent manner. These observations can be translated into phenotypic effects that the depletion or pharmacological inhibition of USP2 effectively sensitized ErbB2-positive breast cancer cells to HSP90 inhibition.

To date, reversible ubiquitylation has been implicated in the endocytic regulation of diverse cell surface proteins, suggesting a generic means employed by cells to dynamically control the abundance of various proteins residing in cell membranes. Of note, only a handful of deubiquitylases have so far been associated with the endocytic pathway, which are recurrently exemplified by AMSH, USP8, and USP2, hence suggesting promiscuous DUB activity on endo-lysosomes. Interestingly, deubiquitylases can lead to either positive or negative regulation upon the endocytic degradation of cell surface proteins, primarily depending on locations of DUB action [44]. Receptor deubiquitylation occurring on early endosomes frequently promotes the recycling of receptors to the surface, while DUB on late endosomes/lysosomes retrieves ubiquitin molecules and facilitates lysosomal degradation of committed cargoes [25, 27]. Regarding USP2, investigations have hitherto demonstrated its protective effects toward several endocytic substrates, including the epithelial sodium channel ENaC, the parathyroid hormone receptor PTHR, the potassium channel KCNQ1, EGFR and the low-density lipoprotein receptor LDLR, suggesting USP2 as a common endocytosis-associated DUB [45–49].

It has been well-established that the molecular chaperone function of HSP90 is crucial to maintain the stability of a wide spectrum of cellular proteins, including many clients that are closely implicated in tumorigenesis such as ErbB2, the serine/threonine-protein kinase RAF, protein kinase B (PKB/AKT), and cyclin-dependent kinase 4 (CDK4) [34]. Accordingly, HSP90 has long been considered as a top therapeutic target in cancer treatment. It nevertheless turned out to be disappointing following a series of clinical trials that the efficacy of HSP90 inhibition was revealed to be moderate and so far none HSP90 inhibitor succeeded in clinical application to receive an FDA approval, albeit with decades of investigations [33]. To improve the therapeutic effects of HSP90-targeted approach, alternative strategies have been developed, including inhibitor conjugates that comprise of HSP90 inhibitor and chemotherapeutic agents, as well as combined therapies to simultaneously target HSP90 and kinases [50, 51]. Based on findings from the present investigation, we propose a novel therapeutic strategy to treat ErbB2-positive breast cancer via combined pharmacological inhibition of HSP90 and USP2. Through promoting ErbB2 ubiquitylation, USP2 inhibitor is expected to augment the pro-degradation effects of HSP90 inhibitors toward ErbB2 within their respective therapeutic windows, thus eliciting potent anti-tumor effects with tolerable adverse side effects, which warrants further investigations and clinical evaluations.

## Supplementary information

Legends for Supplementary Figures and Table

Supplementary Figure 1

Supplementary Figure 2

Supplementary Figure 3

Supplementary Figure 4

Supplementary Figure 5

Supplementary Figure 6

Supplementary Table 1
