# Supplementary material for: The deubiquitylase USP2 maintains ErbB2 abundance via counteracting endocytic degradation and represents a therapeutic target in ErbB2-positive breast cancer
Source: Cell Death Differ. 2020 Apr 23;27(9):2710–25. doi: 10.1038/s41418-020-0538-8 (PMC7429833; doi:10.1038/s41418-020-0538-8)
Supplement: Supplementary file 1 — Legends for Supplementary Figures and Table [file 41418_2020_538_MOESM1_ESM.docx]

**Supplementary information:**

**The deubiquitylase USP2 maintains ErbB2 abundance via counteracting endocytic degradation and represents a therapeutic target in ErbB2-positive breast cancer**

Jinrui Zhang^1#^, Shuyan Liu^1#^, Qiong Li^1^, Yulin Shi^1^, Yueguang Wu^1^, Fang Liu^1^, Shanshan Wang^1^, Mohamed Y. Zaky^1, 2^, Waleed Yousuf^1^, Qianhui Sun^1^, Dong Guo^1^, Taishu Wang^1^, Yingqiu Zhang^1^, Yang Wang^1^, Man Li^1, 3^*, Han Liu^1^*

Legends for 6 Supplementary Figures and 1 Supplementary Table

Supplementary Figure 1. 17-AAG induces the direct ubiquitylation of ErbB2. Cultured SKBR3 cells were treated with 17-AAG at 500 nM for indicated times and lysed using 2% SDS-containing buffer. ErbB2 proteins were immunoprecipitated using mouse anti-ErbB2 antibody (clone 9G6) under denatured conditions. A and B show immunoblotting analyses of cell lysates (input) and immunoprecipitation (IP) samples using indicated antibodies, respectively. C, quantification of ubiquitin signal after ErbB2 normalization. All error bars represent standard error of the mean (n = 3).

Supplementary Figure 2. Colocalizations of internalized ErbB2 with endo-lysosomal markers following HSP90 inhibition. A, B, and C, AU565, HCC1419, and ZR-75-30 cells as indicated were treated with 500 nM of 17-AAG for 4 h, prior to immunofluorescence analysis using anti-ErbB2 and either anti-EEA1 or anti-LAMP1 antibodies in parallel with untreated control cells. Representative confocal sections are shown with magnified insets to show colocalizations. Scale bar = 10 μm.

Supplementary Figure 3. Internalized ErbB2 incurred by 17-AAG treatment accumulates on lysosomes following chloroquine treatment. A, B, and C, AU565, HCC1419, and ZR-75-30 cells as indicated were treated with chloroquine (CQ, 100 μM) for 30 min before 17-AAG addition (500 nM) for 4 h. Cells were processed for immunofluorescence and confocal microscopy with anti-ErbB2 and anti-LAMP1 antibodies. Representative images show confocal sections with magnified insets demonstrating colocalizations. Scale bar = 10 μm.

Supplementary Figure 4. DUB knockdown screen reveals USP2 as a regulator of ErbB2 expression. A, cultured SKBR3 cells stably expressing shRNAs targeting specific DUBs were lysed for immunoblotting assays to probe for ErbB2. Vinculin was detected to confirm equal loading. B, quantification of ErbB2 levels from A. C, SKBR3 stable cell lines as indicated were treated with DMSO as control or 17-AAG at 80 nM for 10 h before lysis. ErbB2 expression was inspected by immunoblotting. Vinculin blots show equal loading. D, quantification of the levels of ErbB2 in 17-AAG treated cells relative to control-treated counterparts. Error bars represent standard error of the mean (n = 3), with ** indicating p < 0.01.

Supplementary Figure 5. Validation of stable cell lines. A, SKBR3 cells with stable transfection of pLKO.1 constructs expressing shRNAs targeting USP2, AMSH, AMSHLP, and USP8 (two shRNAs per target) were harvested and total RNA was extracted. mRNA levels of four DUBs in stable shRNA expressing cells were compared to empty vector control group (pLKO.1) as indicated by performing quantitative RT-PCR analysis. B, cultured AU565, AU565-USP2del, and AU565-USP2del-CCND1 cells were lysed and protein samples were subjected to immunoblotting analysis with indicated antibodies. Actin was probed to show equal loading. Column chart on the right shows relative quantification of ErbB2 expression in 3 cell lines. C, 4T1, 4T1-pCDH (empty vector control), and 4T1-ErbB2 stable cells were lysed for immunoblotting analysis with indicated antibodies to confirm ErbB2 expression. Error bars represent standard error of the mean (n = 3), with ** indicating p < 0.01.

Supplementary Figure 6. The effects of DUB inhibitors on ErbB2 turnover and ubiquitylation. A and B, SKBR3 and HCC1954 cells were treated with cycloheximide (100 μg/ml) in the presence of IU1 (20 μM), P5091 (20 μM), or ML364 (10 μM) for indicated times (DMSO as control). Cell lysates were analyzed by immunoblotting with indicated antibodies. Vinculin was probed as loading control. Below column charts show relative quantiﬁcation of ErbB2 levels. C, AU565 cells were treated with DMSO, IU1 (20 μM), P5091 (20 μM), or ML364 (10 μM) for 12 h. Cell lysates were prepared and ErbB2 was immunoprecipitated using mouse anti-ErbB2 antibody (clone 9G6). Cell lysate (input) and immunoprecipitation (IP) samples were subjected to immunoblotting assays with indicated antibodies. Column chart on the right shows the quantification of relative ubiquitin signal after ErbB2 normalization. D, AU565 cells were treated with 17-AAG at 80 nM in the presence of IU1 (20 μM), P5091 (20 μM), or ML364 (10 μM) as indicated for 12 h. ErbB2 was immunoprecipitated using mouse anti-ErbB2 antibody (clone 9G6) from cell lysates and processed for immunoblotting analysis along with cell lysates. Column chart on the right shows the relative quantification of ubiquitylation after ErbB2 normalization. Error bars represent standard error of the mean (n = 3).

Supplementary Table 1. List of target sequences for shRNAs used in this study.
