# Supplementary figures and images for: The deubiquitylase USP2 maintains ErbB2 abundance via counteracting endocytic degradation and represents a therapeutic target in ErbB2-positive breast cancer
Source: Cell Death Differ. 2020 Apr 23;27(9):2710–25. doi: 10.1038/s41418-020-0538-8 (PMC7429833; doi:10.1038/s41418-020-0538-8)

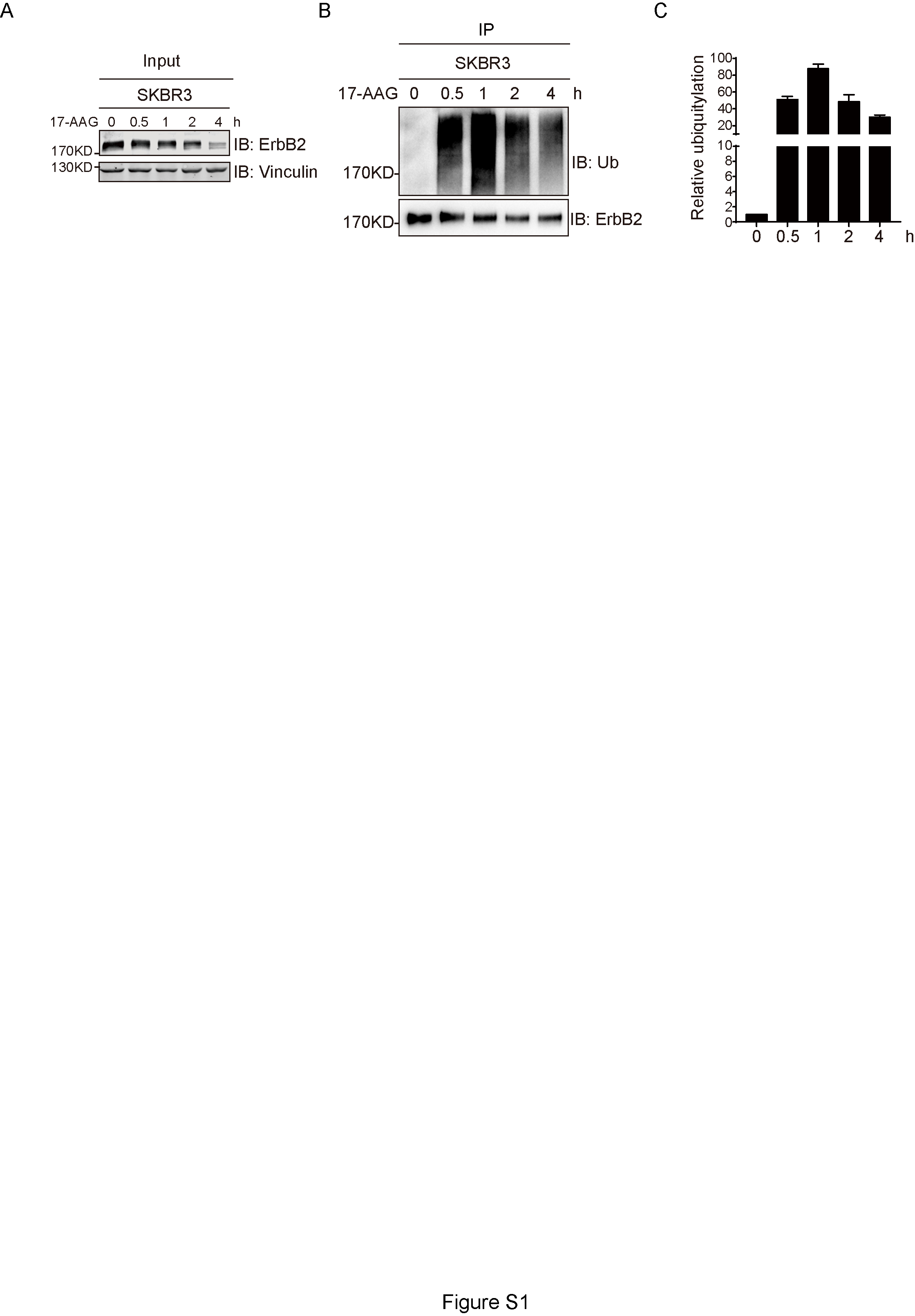

Supplement: Supplementary file 2 — Supplementary Figure 1 [file 41418_2020_538_MOESM2_ESM.tif]

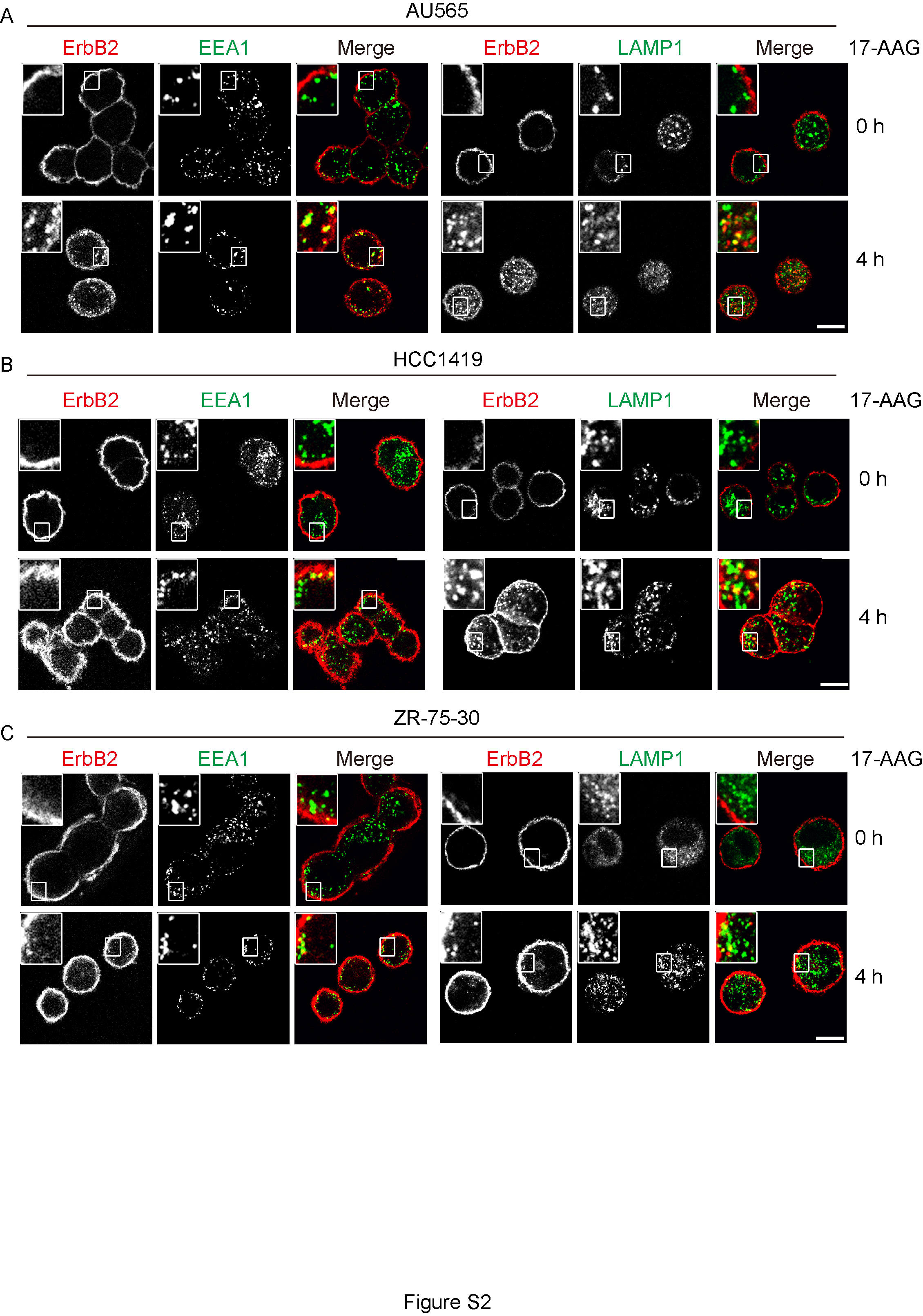

Supplement: Supplementary file 3 — Supplementary Figure 2 [file 41418_2020_538_MOESM3_ESM.tif]

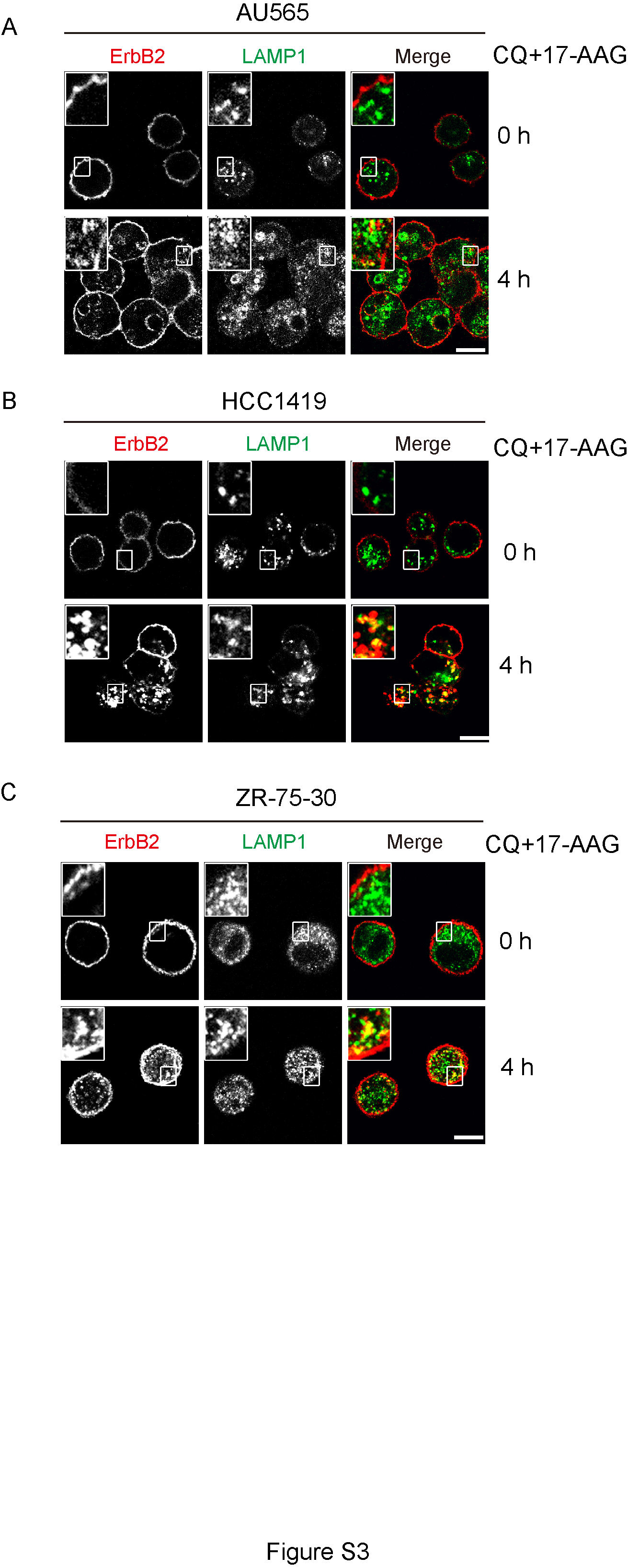

Supplement: Supplementary file 4 — Supplementary Figure 3 [file 41418_2020_538_MOESM4_ESM.tif]

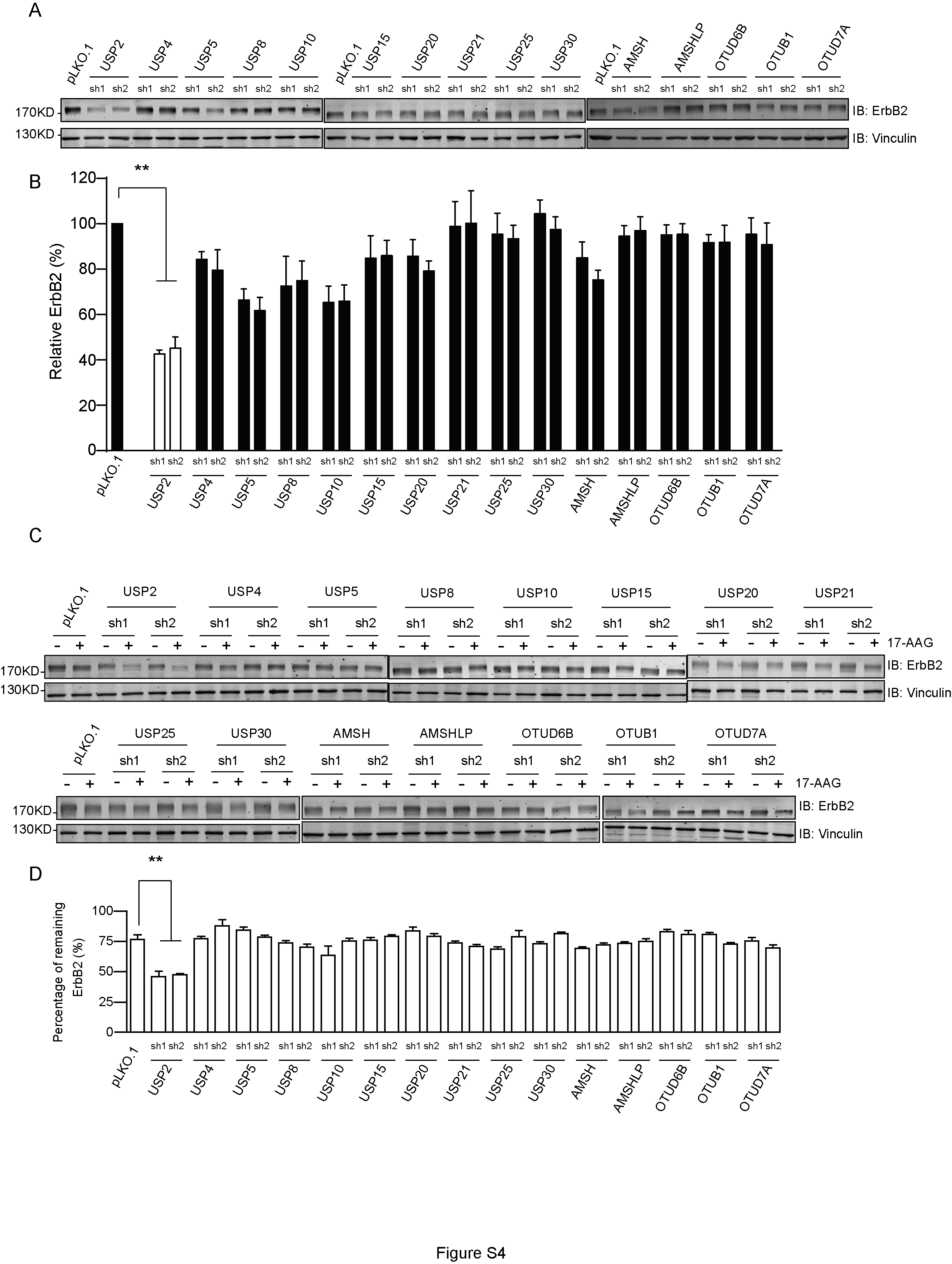

Supplement: Supplementary file 5 — Supplementary Figure 4 [file 41418_2020_538_MOESM5_ESM.tif]

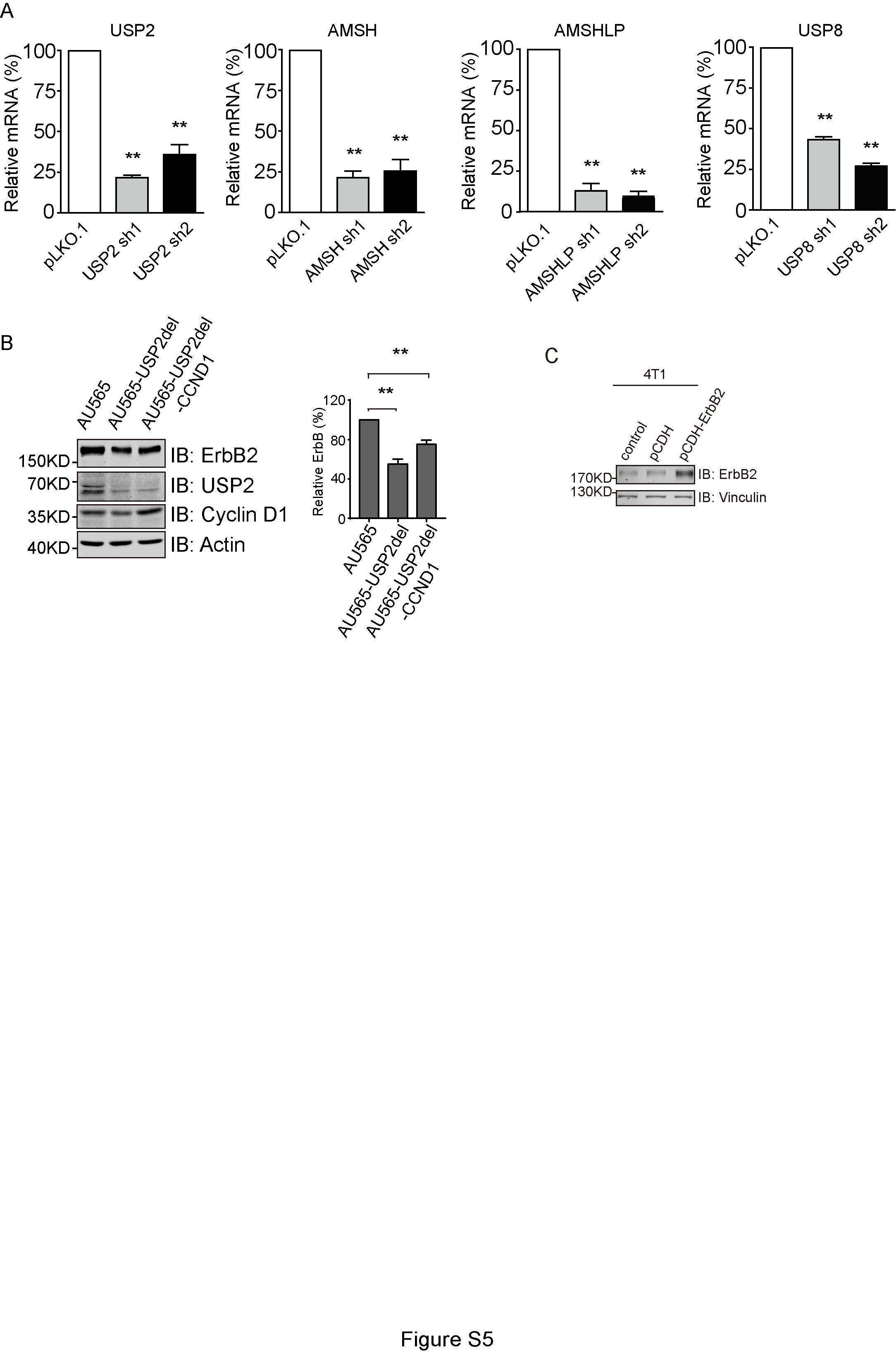

Supplement: Supplementary file 6 — Supplementary Figure 5 [file 41418_2020_538_MOESM6_ESM.tif]

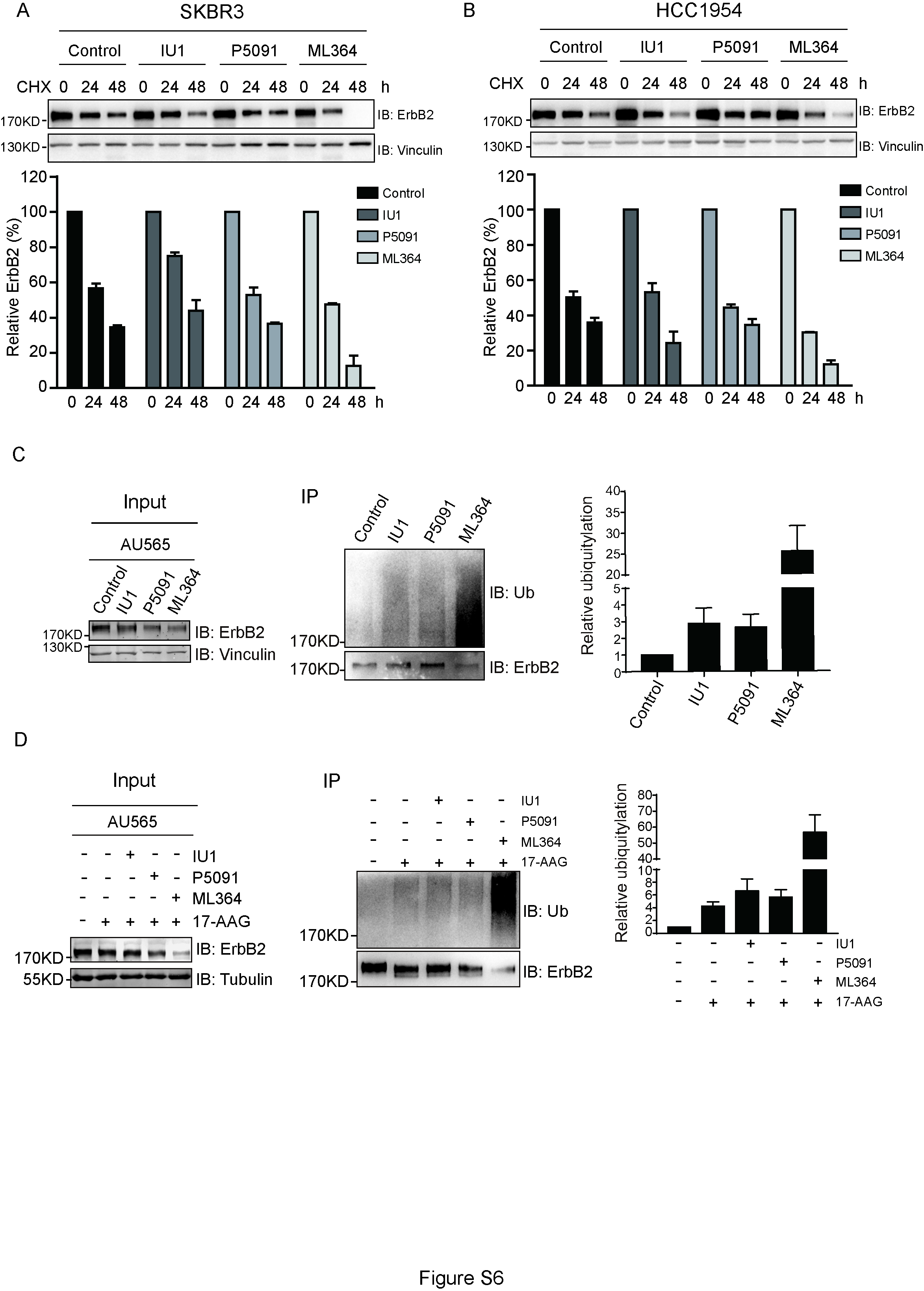

Supplement: Supplementary file 7 — Supplementary Figure 6 [file 41418_2020_538_MOESM7_ESM.tif]
