# Supplementary material for: The deubiquitylase USP2 maintains ErbB2 abundance via counteracting endocytic degradation and represents a therapeutic target in ErbB2-positive breast cancer
Source: Cell Death Differ. 2020 Apr 23;27(9):2710–25. doi: 10.1038/s41418-020-0538-8 (PMC7429833; doi:10.1038/s41418-020-0538-8)
Supplement: Supplementary file 8 — Supplementary Table 1 [file 41418_2020_538_MOESM8_ESM.docx]

Supplementary Table 1. List of target sequences for shRNAs used in this study.

| *USP2* | *sh1* | CCCATTGCTAAGCGAGGTTAT |
| --- | --- | --- |
|  | *sh2* | GCTCACAACATTTGTGAACTT |
| *USP4* | *sh1* | CATGTCCGAGTTTGTCTGTAA |
|  | *sh2* | GCACCACTGACTGACTACTTT |
| *USP8* | *sh1* | GCTGTGTTACTAGCACTATAT |
|  | *sh2* | CCACAGATTGATCGTACTAAA |
| *USP5* | *sh1* | AAGAGATTCACTAAGAACA |
|  | *sh2* | CAGAACAGAAGGAAGTTCA |
| *USP13* | *sh1* | CGATTTAAATAGCGACGATTA |
|  | *sh2* | GCCAGTATCTAAATATGCCAA |
| *USP10* | *sh1* | CCCATGATAGACAGCTTTGTT |
|  | *sh2* | GCTGTGGATAAACTACCTGAT |
| *USP20* | *sh1* | GCGACCATCATCAGGATCAAA |
|  | *sh2* | CGACACCTTCATCAAGTTGAA |
| *USP21* | *sh1* | GACCCTCTGCAATATCACTTT |
|  | *sh2* | CCACTTTGAGACGTAGCACTT |
| *USP25* | *sh1* | GCTGTAGAAGATATGAGAAAT |
|  | *sh2* | GCACTTCTCCTGTTGACGATA |
| *USP30* | *sh1* | CACACCAGTATTTATCCTTAA |
|  | *sh2* | CCATGTCATTACCTCGTCATT |
| *AMSH* | *sh1* | GCAATATGAATGGAGCTTATT |
|  | *sh2* | CCAGAGTCAGTAGCCATTGTT |
| *AMSHLP* | *sh1* | GCTTGAGGTTTCTGCTTGTAA |
|  | *sh2* | GCTATGCCTGACCATACAGAT |
| *OTUB1* | *sh1* | AGGAGTATGCTGAAGATGACA |
|  | *sh2* | CACCACCAATCCGCACATCTT |
| *OTUD6B* | *sh1* | GCTGACTACTAAGGAGAATAA |
|  | *sh2* | CGATGAGACTAATGCAGTGAA |
| *OTUD7A* | *sh1* | CCATCGTTGTTGTGGCAGATA |
|  | *sh2* | CGAGGATTTCAGGAGCTTCAT |
